# Review of Statistical Methodologies for Detecting Drug–Drug Interactions Using Spontaneous Reporting Systems

**DOI:** 10.3389/fphar.2019.01319

**Published:** 2019-11-08

**Authors:** Yoshihiro Noguchi, Tomoya Tachi, Hitomi Teramachi

**Affiliations:** ^1^Laboratory of Clinical Pharmacy, Gifu Pharmaceutical University, Gifu, Japan; ^2^Laboratory of Community Healthcare Pharmacy, Gifu Pharmaceutical University, Gifu, Japan

**Keywords:** pharmacovigilance, statistical methodology, signal detection, spontaneous reporting systems, drug–drug interaction

## Abstract

Concomitant use of multiple drugs for therapeutic purposes is known as “polypharmacy situations,” which has been recognized as an important social problem recently. In polypharmacy situations, each drug not only induces adverse events (AEs) but also increases the risk of AEs due to drug–drug interactions (DDIs). The proportion of AEs caused by DDIs is estimated to be around 30% of unexpected AEs. The randomized clinical trials in pre-marketing typically focus emphasis on the verification of single drug safety and efficacy rather than the surveys of DDI, and therefore, patients on multiple drugs are usually excluded. However, unlike pre-marketing randomized clinical trials, in clinical practice (= post marketing), many patients use multiple drugs. The spontaneous reporting system is one of the significant sources drug safety surveillance in post-marketing. Commonly, signals of potential drug-induced AEs detected from this source are validated in real-world settings. Recently, not only methodological studies on signal detection of “single” drug, but also on several methodological studies on signal detection of DDIs have been conducted. On the other hand, there are few articles that systematically summarize the statistical methodology for signal detection of DDIs. Therefore, this article reviews the studies on the latest statistical methodologies from classical methodologies for signal detection of DDIs using spontaneous reporting system. This article describes how to calculate for each detection method and the major findings from the published literatures about DDIs. Finally, this article presented several limitations related to the currently used methodologies for signal detection of DDIs and suggestions for further studies.

## Introduction

For safety surveillance of a drug, several data-mining algorithms are used to detect quantitative signals from spontaneous reporting systems. The data-mining algorithms include the frequency statistical models are the *proportional reporting ratio* (*PRR*) (Evans et al., 2001) and the *reporting odds ratio* (*ROR*) (van, Puijenbroek et al. 2002), and the Bayesian statistical models are [the *information component* (*IC*) as the *Bayesian Confidence Propagation Neural Network* (*BCPNN*) (Bate et al., 1998) and the *gamma-Poisson shrinker* (*GPS*) (Szarfman et al., 2002) used as the empirical Bayes geometric mean (*EBGM*)].

Although, the recent extension of the *IC* and the *GPS* can accommodate signals of high-order interactions (Almenoff et al., 2003; Yang and Fram, 2004; Norén et al., 2006; DuMouchel and Harpaz, 2012), generally, the *PRR* and the *ROR* are exploited for early signal detection of unknown “single” drug-induced adverse events (AEs). And these detection models might detect potential drug-induced AEs that could not be found clinical trials of pre-marketing using spontaneous reporting systems including post-marketing data.

The randomized clinical trials in pre-marketing typically focus emphasis on the verification of single drug safety and efficacy rather than the surveys of drug–drug interactions (DDIs), and therefore patients on multiple drugs are usually excluded from the clinical trial. However, unlike pre-marketing randomized clinical trials, in clinical practice (= post marketing), many patients use multiple drugs, as in polypharmacy situations.

Concomitant use of multiple drugs can affect the biological action of the related drugs. The main types of DDIs include pharmacokinetic and pharmacodynamic interactions (Aronson, 2004). Of them, the pharmacokinetic interactions might affect the metabolism of drug that determine bioavailability. On the other hand, there is no change in blood levels of drugs in the pharmacodynamic interactions, which can occur either competitively or non-competitively at the pharmacological receptor level.

In concomitant use of multiple drugs, each drug not only induces AEs but also increases the risk of AEs due to DDIs. The proportion of AEs caused by DDI has been estimated to be around 30% of unexpected AEs (Pirmohamed and Orme, 1998).

Adverse events caused by DDIs can also be prevented if discovered early like single drug-induced AEs, and it is practically difficult to examine the interactions of all drug combinations in the pre-marketing stage (Banda et al., 2016). Therefore, post-marketing surveys will help early detection of unknown AEs not only caused by single drug but also DDIs.

Recently, several methodological studies on signal detection of DDIs have been conducted. Herein, we review studies on the statistical methodologies for signal detection of DDIs using spontaneous reporting systems.

## Statistical Methodology

### Logistic Regression Model

van Puijenbroek et al. proposed a statistical method using the *logistic regression model* for detecting signals of DDIs from a spontaneous reporting system (van, Puijenbroek et al. 1999; van, Puijenbroek et al. 2002).

The *ROR* is a statistical model similar to odds ratio (van, Puijenbroek et al. 2002), and using the *logistic regression model* shown in Eq. 1, the *ROR* adjusted for age, gender, and concomitant drugs (*drug D*_1_ and *drug D*_2_) is used as the *adjusted ROR*.

(1) $$log\left(odds\right)=\beta_{0}+\beta_{1}\;a+\beta_{2}\;G+\beta_{3}\;x_{1}+\beta_{4}\;x_{2}+\beta_{5}\;x_{1}\;x_{2\;}\;$$

where, *a* = age, *G* = gender, *x*_1_ = *drug D*_1_, *x*_2_ = *drug D*_2_, and *x*_1_ *x*_2_ = the concomitant use of *drug D*_1_ and *drug D*_2_.

In their first study, the authors showed that concomitant use of oral contraceptives and the antifungal itraconazole resulted in the occurrence of withdrawal bleeding. In the second study, the authors showed that the efficacy of diuretics decreased with the concomitant use of diuretics and non-steroidal anti-inflammatory drugs, resulting in worsening of congestive heart failure (van, Puijenbroek et al., 1999).

Signal detection using the *logistic regression model* has some limitations (e.g., ignoring dependencies/associations between AEs and regression analysis of more than 10,000 drugs as included in a spontaneous reporting system).

To overcome the limitations of the *classical logistic regression model*, a new statistical model; the *Bayesian logistic regression model*, which extended the logistic regression model corresponding to data of very large dimensions, was proposed. The *Bayesian logistic regression model* can perform regression analysis using millions of predictors contained in a spontaneous reporting system. (Genkin et al., 2007).

Using the *Bayesian logistic regression model*, Caster et al. also addressed masking effect (*cf. Limitation*) that affects background reporting of AEs (Wang et al., 2010) and confounding caused by the concomitant use of multiple drugs (Caster et al., 2010).

### Extended Gamma-Poisson Shrinker Model

#### Multi-Item Gamma-Poisson Shrinker Model

The *multi-item gamma-Poisson shrinker* (*MGPS*) *model* is currently used by the US Food and Drug Administration (FDA) and is a statistical model that extended the *GPS* model for detecting signals of potential DDIs (Almenoff et al., 2003; Yang and Fram, 2004).

The *MGPS model* can calculate the score of “*Drug*–*Drug*–*Event*” or “*Drug*–*Event*–*Event*” (including that of with higher-order interactions such as “*Drug*–*Drug*–*Drug*–*Event*” or “*Drug*–*Drug*–*Event*–*Event*”). Moreover, the *MGPS model* can be applied to the itemsets of size 3 or more, but as the number of items increases, the calculation amount explosively increases.

In the *MGPS model*, *Excess2* is used an indicator value. The signal detection threshold value is not set, and as the value of *Excess2* is relatively large, the influence of interaction caused by concomitant drugs is predominantly suspected.

For an arbitrary itemset, it is desirable to estimate the expectation λ = *E* [*N/E*]. Where, *N* is the observed frequency of the itemset (= number of reports) and *E* is the count predicted from an assumption that items are independent, that is, the baseline count.

The observed frequency of itemset is defined by *i*, *j*, *k*,…, as *N* *_i_*, *N* *_j_*, *N* *_k_*…, *E* and other variables are defined as subscript letters as well as *N*. For example, *E* *_ij_* is the baseline prediction for the number of involving items *i* and *j*.

As a common model, baseline counts are calculated based on the assumption of within-stratum independence. *E* calculated under this assumption is often expressed as *E*0.

If all reports are assigned to the strata denoted by *s* = 1, 2,…, S, the proportion of reports in stratum *s* that contain the item *i* is expressed by *P* *_i_* *^s^*, and the total number of reports in stratum *s* is expressed by *n* *_s_*.

Here, the frequency of baseline for triple itemset (*i*, *j*, and *k*) is defined under independence as:

(2) $$E0_{ijk}=\underset{}{\sum }n_{s}P_{i}^{s}P_{j}^{s}P_{k}^{s}\;$$

For itemsets of size 3 or more, an “all-2-factor” loglinear model can be defined as the frequency *E*2 for the itemsets that match all the estimated pairwise two-way marginal frequencies but contain no high-order dependencies.

For itemsets of size 3 (e.g., DDI: *drug D*_1_ and *drug D*_2_, and AE), the estimated frequency of the all-2-factor loglinear model can be defined as the frequency *E*2 prediction by simple subtraction is compared.

For example:

(3) $$Excess2_{ijk}=\;\lambda_{ijk}E0_{ijk}-E2_{ijk}\;$$

The parameter λ is estimated by the geometric means, denoted as *EBGM*, of their empirical Bayes posterior distributions.

Detecting the signals of DDIs using the *MGPS model* is based on the *EBGM* value of the two drugs and the lower of the 90% confidence interval (CI) being larger than the upper of the 90% CI estimates for each of the two drugs.

Example, in one of the reports the signals of potential DDIs detected using the *MGPS model* is the AE profile of verapamil (the calcium channel blocker) and the combination of three classes of cardiovascular drugs (Almenoff et al., 2003).

This result revealed that the *MGPS model* for disproportionality measure is a promising statistical model for detecting signals of potential DDIs in polypharmacy situations.

#### Regression-Adjusted Gamma-Poisson Shrinkage Model

The *GPS model* proposed by DuMouchel is worse than the *logistic regression model* (Harpaz et al., 2013). However, unlike the *GPS model*, signal detection using *t*-tests in *logistic regression models* is not suitable for small samples such as rare AEs (DuMouchel and Pregibon, 2001).

DuMouchel et al. proposed the *Regression-adjusted gamma-Poisson shrinkage* (*RGPS*) model, which integrated the *GPS model* and the *logistic regression model* into a hybrid detection model with the advantages of both, to overcome the disadvantages of the *GPS* model (DuMouchel and Harpaz, 2012).

The *RGPS model* is similar to the *MGPS model* (cf. *Multi-item Gamma-Poisson Shrinker Model*) in that the relative reporting rate (*RRR*) is entered into the *Bayesian gamma-Poisson shrinking algorithm*, and a reliable estimate rate and CI are obtained.

On the contrary, the major difference between the *RGPS model* and the *MGPS model* is that the *MGPS model* do not consider the effects for polypharmacy, and thus may lead to the underestimation of disproportionality estimate for the drug of interest. In addition, the *RGPS model* can handle this question.

Additionally, the values of the adjusted expected value (E) in *the RGPS model* is not calculated by standard logistic regression but instead the extended logistic regression.

It is recommended to replace *EBGM* as the posterior geometric mean with the *empirical Bayes relative reporting ratio* (*EBRRR*) as the posterior mean in the *RGPS model*.

For each response, the (*N* *_j_*, *E* *_j_*) pairs from the previous step are input into a gamma-Poisson shrinkage algorithm. The prior distributions are assumed to be simple gamma distributions rather than a mixture of two gamma distributions as is done in the *MGPS model*. Specifically, a two-parameter gamma Poisson model is used to produce shrinkage estimates, where the prior distribution of the relative reporting ratios is assumed to be Gamma (*γ*, *δ*) and where the (*N* *_j_*, *E* *_j_*) pairs are used to estimate the hyperparameters *γ* and *δ*. The posterior mean of a drug relative reporting ratio is then *EBRRR* *_j_* = (*N*_j_ + *γ*)/(*E* *_j_* + *δ*), and *RRR*05 and *RRR*95 are computed using the appropriate gamma distribution Gamma(*N*_j_ + *γ*, *E* *_j_* + *δ*) (DuMouchel and Harpaz, 2012).

In the *RGPS model* of DDIs, *n* *_jk_* is defined as the number of reports including both *drug* *_j_* and *drug* *_k_*, and *N* *_jk_* is defined as the number of reports related to expected AEs. Then, *EBRRR* *_j_* and *EBRRR* *_k_* are defined as the corresponding disproportionality estimates for the two drugs in report *i*.

(4) $$p_{i}=\;P_{\alpha }\left(\mu_{i}=\;\beta_{0_{g\left(i\right)}}+\;\underset{}{\sum }X_{ij}\beta_{j}\right)\;$$

where *P* *_α_* is the function that links the linear predictor *µ* *_i_* to the probability scale and *β* *_j_* and *β*_0_ *_g_*_(_ *_i_*_)_ are the estimated coefficients for the drugs and intercepts, where the intercept depends on which grouped-stratum *g*(*i*) report *i* belong to. Additionally, Let *X* *_ij_* = 1 if drug *j* is included in report *i*, *X* *_ij_* = 0 otherwise, and let *N* *_j_* be the number of events reported with drug *j*.

*E* *_jk_* is defined as the expected value (*E*) of *N* *_jk_* under the null hypothesis that both *drug* *_j_* and *drug* *_k_* have no effect of the *RRR*.

(5) $$E_{jk}=\underset{}{\sum }X_{ij}X_{ik}P_{\alpha }\left(\mu_{i}-\beta_{j}-\beta_{k}\right)\;\left(1\leq j<k\leq J_{\mathrm{int}}\right)\;$$

where, *β* *_j_* or *β* *_k_* is considered as 0 if the suspected drug was not in the *logistic regression model*.

“No interaction” indicates that the disproportionality measure for both the drugs (= *N* *_jk_*/*E* *_jk_*) is expected to be higher for the *EBRRR* *_j_* and *EBRRR* *_k_*. Therefore, the no-interaction expected count is defined as follows:

(6) $$E_{jk}^{*}=E_{jk}^{*}\text{max}\left(EBRRR_{j},\;EBRRR_{k}\right)\;$$

There will be *J*_int_ (*J*_int_ – 1)/2 raw interaction ratio (*INTRR* *_jk_*) of the form:

(7) $$INTRR_{jk}=\frac{N_{jk}}{E_{jk}^{*}}$$

DuMouchel et al. proposed a method to use one-parameter prior gamma distribution (γ_1_, γ_1_), of mean 1, as a model for the mean of *INTRR* *_jk_*, and estimate γ_1_ by inputting the set (*N* *_jk_*_,_ *E* ^*^ *_jk_*) into the empirical Bayes estimation.

As a result, the posterior mean of the interaction ratio is expressed as follows:

(8) $$INTEB_{jk}=\frac{N_{jk}+\;\gamma_{1}}{E_{jk}^{*}+\;\gamma_{1}}\;$$

The posterior 5% limit (*INTEB*_05_ *_jk_*) and posterior 95% limit (*INTEB*_95_ *_jk_*) are the corresponding quantiles of the gamma distribution (*N* *_jk_* + *γ*_1_, *E* ^*^ *_jk_* + *γ*_1_).

The proposed *RGPS model* only presents interaction estimates if *INTEB*_05_ > *INTEB*_05min_ or *INTEB*_95_ < *INTEB*_95max_ with the default values *INTEB*_05min_ = 1 and *INTEB*_95max_ = 1/3 by DuMouchel and Harpaz (2012).

If the *INTEB* is very low, it has not yet been completely verified whether it are the signals of potential DDIs. However, because such results are often obtained, the further verification will be necessary.

### Extended Information Component Model

The *IC* is a measure of association of pairs of drug and AEs only, but there is often an interest in high-order interactions as DDIs (= itemsets of size 3).

Although, an extension of the *IC* to 3rd-order associations including 3 itemset as DDIs was proposed by Orre et al. (2000), the proposed method did not compensate for pairwise associations. Therefore, Norén et al. (2006) proposed the following definition for the *extended IC model*:

(9) $$IC_{xyz}=IC_{xy|z}-IC_{xy}=IC_{yz|x}-IC_{yz}$$

where,

(10) $$IC_{xy|z}=log_{2}\frac{P\left(xy|z\right)}{P\left(x|z\right)P\left(y|z\right)}$$

As with simple algebraic operations, *IC* *_xyz_* can be re-expressed as follows:

(11) $$\begin{array}{c} IC_{xyz}=log_{2}\frac{P\left(yz|x\right)}{P\left(y|x\right)P\left(z|x\right)}\;-log_{2}\frac{P\left(y\right)P\left(z\right)}{P\left(y,z\right)}\;=\\ log_{2}\frac{P\left(x,y,z\right)P\left(x\right)P\left(y\right)P\left(z\right)}{P\left(x,y\right)P\left(x,z\right)P\left(y,z\right)} \end{array}$$

Although arbitrarily accurate estimates for the posterior mean of *IC* distribution can be used (Koski and Orre, 1998), the maximum *a posteriori* (m.à.p.) estimates can be used for central estimates instead, because *IC* distribution is generally unimodal.

There are three main advantages of the m.à.p. estimate.

First, it is well suited for use in stratified *IC*. Second, it has the intuitive property of being equal to 0 when the estimated joint probability is equal to the product of the estimated marginal probabilities. Third, the concept of most likely value for an unknown parameter is perhaps more natural than that of the expected value.

These are important aspects in drug safety applications, and the results must be interpretable not only by statisticians but also by non-statisticians such as medical professionals.

Norén et al. (2006) proposed the following m.à.p. estimate. Most of the theory developed for the pairwise *IC* model (*IC*_m.à.p._ model) holds approximately the *IC model* for higher order.

In one of the reports, the signals of potential DDIs detected using the *extended IC model* was terfenadine and ketoconazole-induced ventricular fibrillation. There were five reports of ventricular fibrillation due to the combination of terfenadine and ketoconazole in the VigiBase^®^ as a spontaneous reporting system, and the extended *IC* (*IC* *_xyz_* ** *_m.à.p._*) value is 2.40 with the lower of the 95% CI of 1.08 (Norén et al., 2006).

### Ω Shrinkage Measure Model

The *Ω shrinkage measure model* was proposed to calculate the observed-to-expected ratio as a spontaneous reporting system for detecting the signals of potential DDIs (Norén et al., 2008).

Norén et al. criticized the *logistic regression model* in missing out on several signals that strongly suggestive of potential DDIs, additionally, they demonstrated that after conducting comparative studies using the World Health Organization database, the *Ω shrinkage measure model* is a refined method compared to the *logistic regression model*.

**Figure 1:**
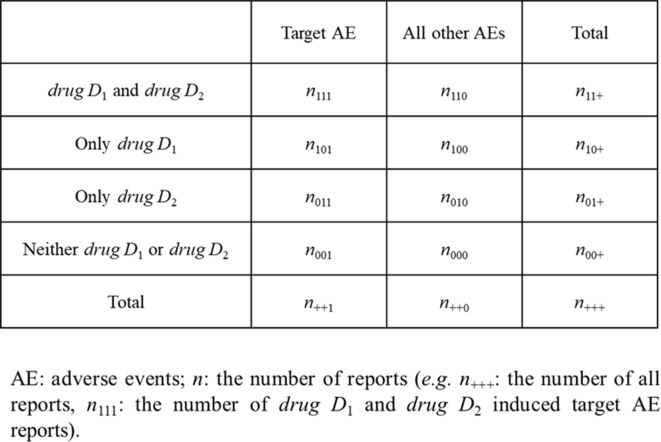
Four-by-two contingency table for the evaluation of drug–drug interaction.

For the *Ω shrinkage measure model*, the observed reporting ratio was *f*_11_ of AE caused by concomitant use of 2 drugs: *drug D*_1_ and *drug D*_2_, in addition, its expected value was *E*[*f*_11_].

(12) $$f_{00}=\frac{n_{001}}{n_{00+}},\;\;f_{10}=\frac{n_{101}}{n_{10+}},\;\;f_{01}=\frac{n_{011}}{n_{01+}},\;\;f_{11}=\frac{n_{111}}{n_{11+}}$$

where, *n* is the number of reports shown in **Figure 1**. For example, *n*_111_ is the number of reported target AEs caused by *drug D* *_1_* and *drug D* *_2_*.

*E*[*f*_11_] is unknown. However, *f*_11_ can be compared with the estimator *g*_11_ of *E*[*f*_11_], *g*_11_ is given as follows:

(13) $$g_{11}=1-\frac{1}{\max\left(\frac{f_{00}}{1-f_{00}},\;\frac{f_{10}}{1-f_{10}}\right)+\;\max\left(\frac{f_{00}}{1-f_{00}},\;\frac{f_{01}}{1-f_{01}}\right)-\;\frac{f_{00}}{1-f_{00}}\;+1\;}\;$$

When *f*_10_ < *f*_00_ (which denote no risk of AE caused by *drug D*_1_), the most sensible estimator *g*11 = max (*f*_00_, *f*_01_) is yielded and the *vice versa* when *f*_01_ < *f*_00_.

Norén et al. defined a non-shrinkage measure for detecting AEs caused by *drug D*_1_ and *drug D*_2_ as follows:

(14) $${\text{Ω}}_{0}=log_{2}\frac{f_{11}}{g_{11}}$$

However, since the occurrence of AE is rare, *g*_11_ might show very small, and therefore, Ω_0_ is sensitive to spurious relationship and tends to falsely detect a signal.

This is a well-known phenomenon in screening pairwise drug-AE excessive reporting rates in a spontaneous reporting system, and shrinkage has been proven to be an effective approach in reducing the sensitivity to random fluctuations in disproportionality measures based on rare cases. The models such as the *BCPNN* and *EBGM* also used pairwise measures of disproportionality as shrinkage measures.

To construct a similar shrinkage measure from Eq. 14, Norén et al. re-expressed the observed and expected *RRR f*_11_ and *g*_11_ in terms of the observed number of reports *n*_111_ and expected numbers of reports *E*_111_ = *g*_11_×*n*_11+_, respectively:

(15) $$\frac{f_{11}}{g_{11}}=\frac{n_{111}/n_{11+}}{E_{111}/n_{11+}}=\frac{n_{111}}{E_{111}}$$

and proposed the Ω shrinkage measure:

(16) $$\text{Ω}=log_{2}\frac{n_{111}+\alpha }{E_{111}+\alpha }$$

α is the tuning parameter that determines the shrinkage strength. When α = 0, Ω = Ω_0_. The effect of α is equivalent to that of α additional expected reports, and exactly matches the increase in the observed number.

Shrinkage regression can be set as the value of tuning parameter based on cross-validation estimates for classifier performance. However, in a disproportionality analysis, there is no objective basis for selecting a particular value for α. Therefore, in the *Ω shrinkage measure model*, α = 0.5 was set to provide sufficient shrinkage for avoiding disproportional highlighting based on rare reports.

In the frequentist method, Ω differs slightly from Ω_0_ for large *n*_111_ and *E*_111_, and the variance of Ω_0_ is given as follows:

(17) $$\text{Var}\left({\text{Ω}}_{0}\right)=\text{Var}\left(log_{2}\frac{n_{111}}{E_{111}}\right)\approx \frac{1}{n_{111}log{\left(2\right)}^{2}}$$

Using Eq. 17, the lower of the 95% CI for Ω can be estimated using the following equation:

(18) $${\text{Ω}}_{025}=\text{Ω}-\frac{\phi \left(0.975\right)}{log\left(2\right)\sqrt{n_{111}}}$$

where, *ϕ*(0.975) is 97.5% of the standard normal distribution.

On the contrary, in Bayesian method, the exact CI for µ can be obtained as solutions to the following equation, for appropriate posterior quantiles µ_q_:

(19) $$\overset{\mu_{q}}{\underset{0}{\int }}\frac{{\left(E_{111}+\alpha \right)}^{n_{111}+\alpha }}{\Gamma \left(n_{111}+\alpha \right)}u^{n_{111}+\alpha -1}e^{-\left(n_{111}+\alpha \right)u}du=q$$

where, α is the tuning parameter. *n*_111_ and *E*_111_ are the number of reported target AEs caused by *drug D* *_1_* and *drug D* *_2_* and their expected values.

Here, the logarithm of the solution to Eq. 19 for q = 0.025 and 0.975 provides Ω_025_ (the lower limits of 95% CI) and Ω_075_ (the upper limits of 95% CI), respectively.

In both frequentist and Bayesian methods, Ω_025_ > 0 is used as a threshold for detecting the signals of the concomitant use with *drug D*_1_ and *drug D*_2_.

Qian et al. built a computerized system in which data acquisition and placement are automated. The signals of potential DDIs were then detected using this system. (Qian et al., 2010). This study detected the signals of potential DDIs using three different models; the *Ω shrinkage measure model*, the *logistic regression model* (cf*. Logistic Regression Model*), and the *additive model and multiplicative models* (cf*. Additive and Multiplicative Models*). A comparison of signals detected using the three models revealed that the signals of potential DDIs detected on average by at least two models could reflect the fact that the 3 models are highly correlated (Qian et al., 2010).

### Additive and Multiplicative Models

Thakrar et al. (2007) proposed the *additive model* and *multiplicative model* for detecting the signals of potential DDIs. For two models, Thakrar et al. (2007) conducted the retrospective study for detecting the signals of known DDIs using the FDA Adverse Event Reporting System.

**Figure 2:**
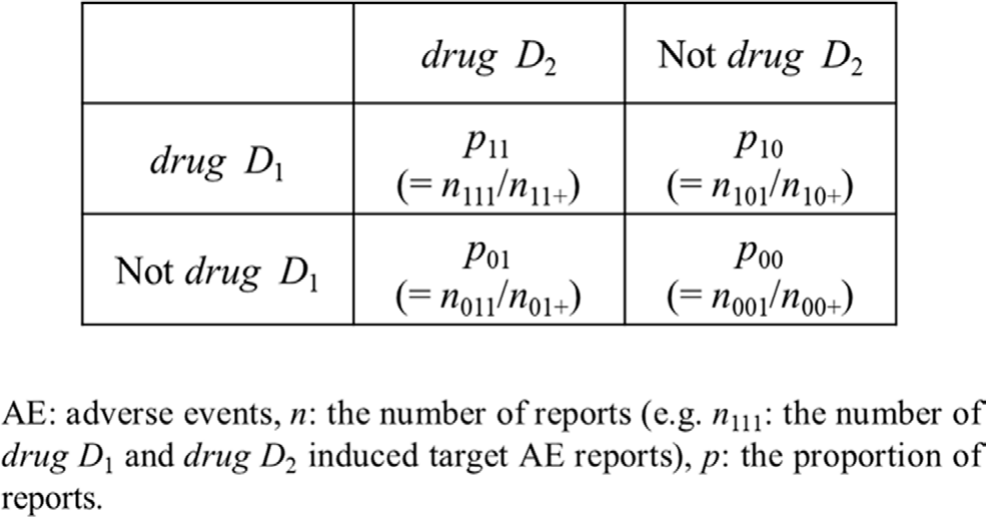
Two-by-two contingency table for the evaluation of drug–drug interaction.

The *additive model* assumes that drug related risks increase additively, on the contrary, the *multiplicative model* assumes that drug related risks increase synergistically. *Additive Model* and *Multiplicative Model* provide the details of each model using **Figure 2**.

#### Additive Model

In the additive model, if the risk associated with *drug D*_1_ without *drug D*_2_ is the same as the risk associated with *drug D*_1_ and *drug D*_2_ together, then there is no signal of DDI. In other words, there are potential DDIs if the combination risk is high compared to what is expected based on the individual drug:

(20) $$p_{11}-p_{00}=\left(p_{10}-p_{00}\right)+\left(p_{01}-p_{00}\right)$$

This equality implies (*RD*: risk difference):

(21) $$RD_{drug\;D_{1}\cap^{\text{​}}\;drug\;D_{2}}=RD_{only\;drug\;D_{1}}+RD_{only\;drug\;D_{2}}$$

That is, when *RD* *_drug_* ** *_D1_*_∩_ *_drug_* ** *_D2_* – *RD* *_only_* ** *_drug_* ** *_D1_* + *RD* *_only_* ** *_drug_* ** *_D2_* > 0 (*p*_11_ − *p*_10_ − *p*_01_ + *p*_00_ > 0), the signal of the *additive model* is detected.

The formal statistical test for DDIs is performed within the framework of binomial distribution linear regression:

$$risk\;of\;event=\alpha_{0}+\alpha_{1}\;x_{1}+\alpha_{2}\;x_{2}+\alpha_{12}\;x_{1}\;x_{2\;}+\varepsilon$$

(22) $$\left(\alpha_{12}=\;p_{11}-p_{10}-p_{01}+p_{00}\right)$$

#### Multiplicative Model

In the *multiplicative model*, under the assumption that the null hypothesis is true (i.e., no interaction), the proportion of an AE associated with the concomitant use of *drug D*_1_ and *drug D*_2_ is the same as the proportional risks of individual drugs in the absence of either *drug D*_1_ or *drug D*_2_.

(23) $$\frac{p_{11}}{p_{00}}=\frac{p_{10}}{p_{00}}\times \frac{p_{01}}{p_{00}}$$

or

(24) $$\frac{p_{11\;}/\;\left(1-p_{11}\right)}{p_{00\;}/\;\left(1-p_{00}\right)}=\frac{p_{10\;}/\;\left(1-p_{10}\right)}{p_{00\;}/\;\left(1-p_{00}\right)}\cdot \frac{p_{01\;}/\;\left(1-p_{01}\right)}{p_{00\;}/\;\left(1-p_{00}\right)}$$

This equality implies:

(25) $$PRR_{drug\;D_{1}\cap^{\text{​}}\;drug\;D_{2}}=PRR_{only\;drug\;D_{1}}\times PRR_{only\;drug\;D_{2}}$$

or

(26) $$ROR_{drug\;D_{1}\cap^{\text{​}}\;drug\;D_{2}}=ROR_{only\;drug\;D_{1}}\times ROR_{only\;drug\;D_{2}}$$

Therefore, if the measure shown in Eq. 27 or Eq. 28 exceeds 1 it can be determined that the signals of potential DDIs are detected. In modeling terminology, the following multiplicative model (Eqs. 25 and 26) can be applied for *log-linear regressions* and *logistic regressions*:

log-linear regressions

$$log\left(risk\;of\;event\right)=\beta_{0}+\beta_{1}\;x_{1}+\beta_{2}\;x_{2}+\beta_{12}\;x_{1}\;x_{2\;}+\varepsilon$$

(27) $$\left(e^{\beta_{12}}=\frac{PRR_{drug\;D_{1}\cap^{\text{​}}\;drug\;D_{2}}}{PRR_{only\;drug\;D_{1}}\times PRR_{only\;drug\;D_{2}}}\right)$$

logistic regressions

$$logit\left(risk\;of\;event\right)=\gamma_{0}+\gamma_{1}\;x_{1}+\gamma_{2}\;x_{2}+\gamma_{12}\;x_{1}\;x_{2\;}+\varepsilon$$

(28) $$\left(e^{\gamma_{12}}=\frac{ROR_{drug\;D_{1}\cap^{\text{​}}\;drug\;D_{2}}}{ROR_{only\;drug\;D_{1}}\times ROR_{only\;drug\;D_{2}}}\right)$$

where, *x*_1_ = *drug D*_1_, *x*_2_ = *drug D*_2_, *x*_1_ *x*_2_ = the concomitant use of *drug D*_1_ and *drug D*_2_.

Thakrar et al. (2007) showed that the *additive model* has higher sensitivity than that of the *multiplicative model* in detecting the signals of potential DDIs. Therefore, Noguchi et al. compared the power of the *additive model* with that of the *multiplicative model* for the *combined risk ratio model* (cf. *Combination Risk Ratio Model*). Similar to the result of Takagi et al., the *additive model* presented higher detection power than that of the *multiplicative model* (sensitivity: 95.62 vs. 65.46%, specificity: 96.92 vs. 98.78%, Youden’s index: 0.925 vs. 0.642, positive predictive value: 89.47% vs. 93.64%, negative predictive value: 98.78 vs. 91.26% *F*-score: 0.924 vs. 0.771) (Noguchi et al., 2018a).

### Combination Risk Ratio Model

To estimate the degree of potential safety risk in combination, Susuta and Takahashi (2014) proposed a risk assessment method for combined use of drugs at a frequency where two or more drugs are reported simultaneously, assuming that the possibility of drug interaction is a combined risk in the occurrence of AEs.

The concomitant use risk was determined when the ratio between the concomitant use indicator and the indicator (e.g., *PRR*, *ROR*) obtained separately for both agents exceeded 2. The following is an expression using the *PRR* as the indicator.

(29) $$Combination\;risk\;ratio=\frac{PRR_{drug\;D_{1}\cap^{\text{​}}\;drug\;D_{2}}}{\text{max}\left(PRR_{drug\;D_{1}},\;PRR_{drug\;D_{2}}\right)}$$

When *n*_111_ ≥ 3, *PRR* *_drug_* ** *_D_*_1__∩_ *_drugD_*_2_ > 2, χ^2^ *_drug_* ** *_D_*_1__∩_ *_drugD_*_2_ > 4, *Combination risk ratio* > 2, it was a signal of DDIs.

The formula for calculating *PRR* and *χ* ^2^ is as follows:

(30) $$PRR=\frac{\left(N_{11}/N_{1+}\right)}{\left(N_{01}/N_{0+}\right)}$$

(31) $$\chi^{2}=\frac{n_{+++}\times {\left(\left|N_{11}\times N_{00}-N_{10}\times N_{01}\right|-n_{+++}/2\right)}^{2}}{N_{1+}\times N_{+1}\times N_{0+}\times N_{+0}}$$

Additionally, to calculate the *PRR* and the *χ* ^2^ of *drug D*_1_ ∩ *drugD*_2_, *drug D*_1_ and *drug D* *_2_*, replace it as follows.

*drug D*_1_ ∩ *drugD*_2_: *N*_11_ = *n*_111_, *N*_00_ = *n*_000_ + *n*_010_ + *n*_100_, *N*_10_ = *n*_110_, *N*_01_ = *n*_001_ + *n*_011_ + *n*_101_, *N*_1+_ = *n*_11+_, *N*_+1_ = *n*_++1_, *N*_0+_ = *n*_00+_ + *n*_01+_ + *n*_10+_, *N*_+0_ = *n*_++0_.

*drug D*_1_: *N*_11_ = *n*_111_ + *n*_101_, *N*_00_ = *n*_000_ + *n*_010_, *N*_10_ = *n*_110_ + *n*_100_, *N*_01_ = *n*_001_ + *n*_011_, *N*_1+_ = *n*_11+_+ *n*_10+_, *N*_+1_ = *n*_++1_, *N*_0+_ = *n*_00+_ + *n*_01+_, *N*_+0_ = *n*_++0_.

*drug D* *_2_*: *N*_11_ = *n*_111_ + *n*_011_, *N*_00_ = *n*_000_ + *n*_100_, *N*_10_ = *n*_110_ + *n*_010_, *N*_01_ = *n*_001_ + *n*_101_, *N*_1+_ = *n*_11+_+ *n*_01+_, *N*_+1_ = *n*_++1_, *N*_0+_ = *n*_00+_ + *n*_10+_, *N*_+0_ = *n*_++0_.

To check the validity of the *combination risk ratio model*, the reports of Stevens–Jonson syndrome (SJS) or toxic epidermal necrolysis caused by the DDIs were analyzed using the Japanese Adverse Drug Event Report database.

As for the concomitant use of suspected drugs, which fulfill the situations of concomitant use risk, SJS: 10 candidates out of 159 combinations and toxic epidermal necrolysis: 22 candidates out of 111 combinations were detected.

In addition, this method proposed by Susuta et al. has been used to search for the DDIs related to the concomitant use of angiotensin receptor blockers and thiazide diuretics combination therapy by Noguchi et al. (2015) and for detecting signals of the concomitant use of deferasirox with other drugs by Mizuno et al. (2016) in Japan.

### Chi-Square Statistics Model

Gosho et al. (2017) proposed the *chi-square statistics model* for detecting the signals of potential DDIs.

First, they developed the following measure (χ_0_) to estimate the discrepancy between the observed and expected numbers of AEs with drug combinations:

(32) $$\;\chi_{0}=\frac{n_{111}-E_{111}}{\sqrt{E_{111}}}$$

The expected number of AEs (*E*_111_) can be estimated using *E*_111_ = *g*_11_·*n*_11·,_ presented in *Ω Shrinkage Measure Model.* The measure χ*_N_*, which is the square root of the chi-square test statistic, is based on the normal approximation of the *Poisson* model, and therefore, χ*_N_* is not suitable for the evaluation of rare events. Thus, when evaluating rare events, it is generally considered more appropriate to use the chi-square test with Yate’s correction than the standard chi-square test (Yates, 1934), hence, χ was also corrected with the correction term “0.5” based on the chi-square test with Yate’s correction:

(33) $$\;\chi =\frac{n_{111}-E_{111}-0.5}{\sqrt{E_{111}}}$$

Gosho et al. (2017) set χ > 2 and χ > 2.6 as thresholds for detecting the signals of AEs caused by DDIs in a simulation study. These cutoff values are specified based on 95% and 99% of chi-square distribution with one degree of freedom. According to this simulation study, with the criterion: χ > 2, false positives are controlled within acceptable ranges, additionally the *chi-square statistics model* showed higher sensitivity and AUC than those of both frequentist and Bayesian methods of the *Ω shrinkage measure model* (Gosho et al., 2017).

Similar to the *Ω shrinkage measure model*, the detection of signal using the *chi-square statistics model* is designed to focus on the detection of synergistic rather than antagonism among some DDIs.

Gosho (2018) used the *chi-square statistics model* and he *Ω shrinkage measure model* to examine the clinical drug–drug interactions that cause hypoglycemia and rhabdomyolysis (Gosho, 2019).

### Association Rule Mining Model

To comprehensively search for the signals of potential DDIs, if a calculation using the conventional methods that simply create combinations from a large database such as a spontaneous reporting system is used, the considered number of the concomitant use would be enormous. Therefore, it would be difficult to detect the signals of potential DDIs at an early stage.

Contrarily, the *association rule mining model* is frequently used to find interesting combinations hidden in large databases, and not just medical databases. In the *association rule mining model*, the “*a priori algorithm*” can be used to reduce the number of calculations (Agrawal et al., 1993; Agrawal and Srikant,1994).

If the *association rule mining model* was used, it is unnecessary to calculate indicators for all combinations of the concomitant use, as the previous models.

An indicator of a general association rule model is shown below.

Among the transaction *T* as a set of items, an association rule can be expressed as the antecedent of rule *X* → the consequent of rule *Y*; where, *X* and *Y* are mutually exclusive sets of items.

There are several indicators of the *association rule mining model*. First, the *support* is defined as the percentage of all items in both *X* and *Y* to transaction *T* in the data. That is, how frequently the rules (*X* → *Y*) occur within transaction *T*. The *support* is as follows:

(34) $$support\;\left(X\to Y\right)=\frac{\left\{X\;\to Y\right\}}{\left\{T\right\}}$$

Second, the *confidence* is the conditional probability *P*(*Y*|*X*), and measures the reliability of the interference made by the rules (*X* → *Y*). The *confidence* is as follows:

(35) $$confidence\;\left(X\to Y\right)=\frac{support\;\left(X\to Y\right)}{support\;\left(X\right)}$$

Third, the *lift* of an association rule represents the ratio of probability. It is the ratio between the *confidence* of the rule and the *support* of the itemset in the consequent of the rule. The *lift* is as follows:

(36) $$lift\;\left(X\to Y\right)=\frac{confidence\;\left(X\to Y\right)}{support\;\left(Y\right)}=\frac{support\;\left(X\to Y\right)}{support\;\left(X\right)\times support\;\left(Y\right)}$$

If the *lift* is > 1, it shows the degree to which those two occurrences depend on each other. Therefore, the *lift* is often used frequently to assess the interest of a rule.

Finally, the *conviction* of an association rule can be interpreted as the ratio of the expected frequency that *X* occurs without *Y* if *X* and *Y* are independent and divided by the observed frequency of incorrect predictions. The *conviction* is as follows:

(37) $$conviction\;\left(X\to Y\right)=\frac{1-support\;\left(Y\right)}{1-confidence\;\left(X\to Y\right)}$$

In the *lift*, even if *X* and *Y* are interchanged, the value of the indicator is the same. On the contrary, in the *conviction*, when *X* and *Y* are interchanged, the value of the indicator is different. This indicates that the *lift* cannot be evaluated correctly if *Y* is actually the antecedent of rule and *X* is actually the consequent of rule, and the *conviction* can be also evaluated correctly in such a situation.

**Figure 3:**
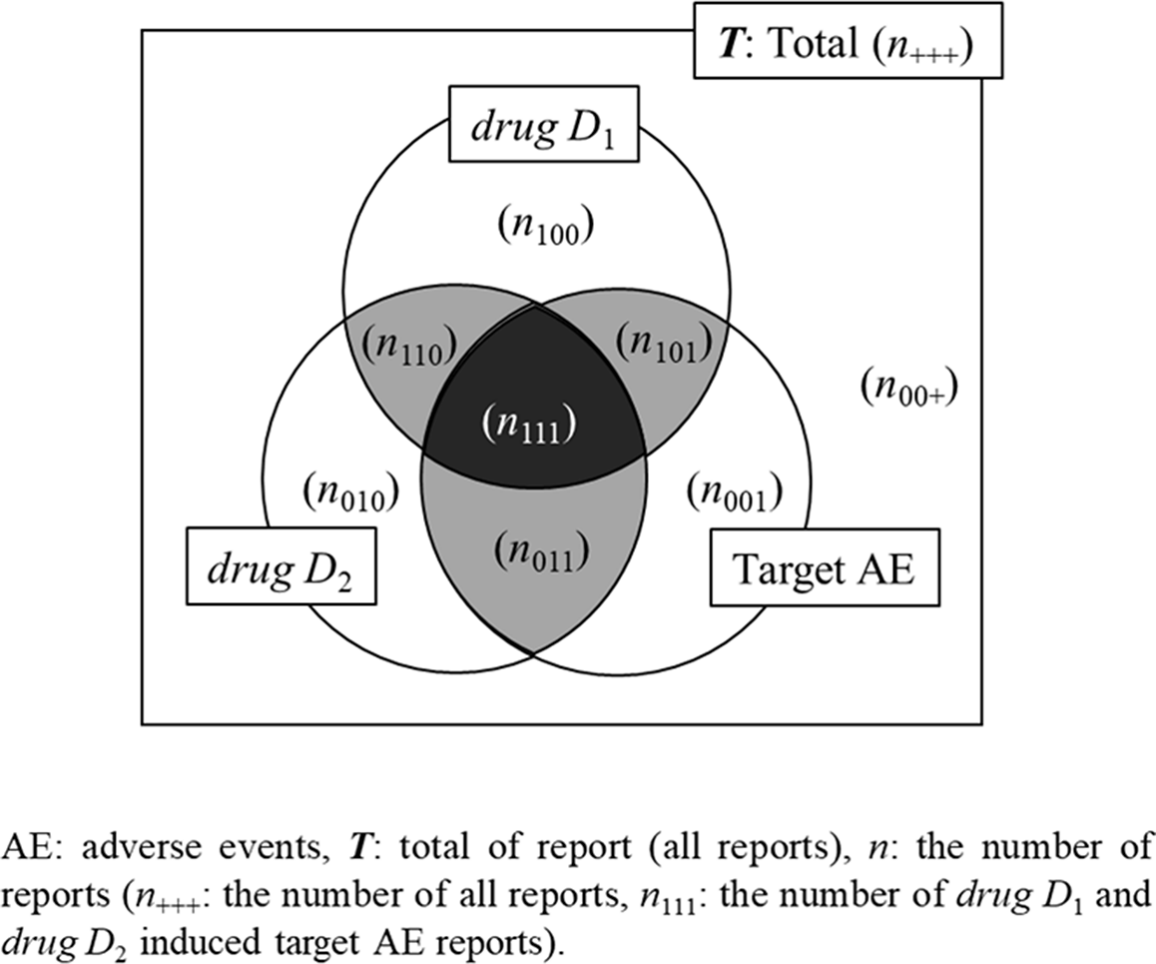
Venn diagram for the evaluation of drug–drug interaction.

So far, we have introduced four indicators that are particularly commonly used in the *association rule mining model*. Next, three search models of the signals of potential DDIs using these indicators are shown using **Figure 3**.

#### Shirakuni’s Method of Association Rule Mining Model

Shirakuni et al. (2009) examined the combined use and discrete use of 2 drugs using *association rule mining model*.

In the combined use of two drugs model, the antecedent of rule *X* was defined as *drugs D*_1_ and *D*_2_, and the consequent of rule *Y* was defined as the target AE (*AE*).

(38) $$\begin{array}{l} support\;\left(drug\;D_{1}\;and\;drug\;D_{2}\to AE\right)=\\ \frac{\left\{drug\;D_{1}\;and\;drug\;D_{2}\to AE\right\}}{\left\{T\right\}} \end{array}$$

(39) $$\begin{array}{l} confidence\;(drug\text{​}D_{1}\;and\;drug\;D_{2}\to AE)=\\ \frac{support(drug\;D_{1}\;and\;drug\;D_{2}\to AE)}{support(drug\;D_{1}\;and\;drug\;D_{2})} \end{array}$$

In the discrete use of 2 drugs model, the antecedent of rule *X* was defined as *drugs D*_1__(or__2)_-induced AE, and the consequent of rule *Y* was defined as *drugs D*_2__(or__1)_. In this rule, both hypotheses and conclusions are relevant to the AE, and therefore, signals can be detected from *drugs D*_1_ and *drugs D*_2_ individually.

The *support* and *confidence* of each drug is calculated for both *drugs D*_1_ and *drugs D*_2_ based on the cases of patients presenting AEs included in the dataset.

(40) support (drug D1(or 2) induced AE→drug D2(or 1))={drug D1(or 2) induced AE→drug D2(or 1)}{T}

(41) confidence (drug D1  and drug D2→AE)=support (drug D1(or2) induced AE→drug D2or1)support (drug D1(or2)induced AE)

Kubota purposed that because the *PRR* show the generation ratio of AEs, the result is evaluated regardless of sample size and χ^2^ is important when examining the total sample size (Kubota, 2001). Therefore, the drugs with high *log PRR* and *log χ* *^2^* values are considered to have a strong signal.

To evaluate *Shirakuni’s method*, the *signal score* obtained by adding the *log PRR* and *log χ* *^2^* was used as the strength of the signal. This *signal score* is also used to compare signals for sex and age differences (Noguchi et al., 2018b; Noguchi et al., 2018c).

(42) $$Signal\;score\;=\;log\;PRR+log\;\chi^{2}$$

The FDA Adverse Event Reporting System dataset had sufficient information to apply the *association rule mining model*. In the *association rule mining model*, high indicators of the *support* and *confidence* are generally evaluated as a strong relationship. Next, Shirakuni et al. (2009) compared each *signal score* of the SJS caused by DDIs with the results of the association rule mining model to evaluate the performance of the proposed model.

In this result, the correlation between “discrete use of 2 drugs” and the *signal score* was weaker than that of “combined use of 2 drugs.” Therefore, it was concluded that, among the two methods of the *association rule mining mode*l proposed by Shirakuni et al. (2009), the method focused on “combined use of 2 drugs” detected such important signals at an early stage.

#### Harpaz’s Method of Association Rule Mining Model

In *Harpaz’s method* (Harpaz et al., 2010), like the combined use of two drugs model suggested by Shirakuni et al. (Shirakuni et al., 2009), the antecedent of rule *X* was defined as drugs D_1_ and D_2_, and the consequent of rule *Y* was defined as the AE.

However, in the *association rule mining model*, it is sometimes inappropriate to evaluate using the *confidence* value. For example, frequently reported AEs (e.g., nausea) produce large confidence values ​​regardless of the drug associated with AEs. Whereas, rarely reported AEs may produce small confidence values, although AEs are strongly associated with certain drugs.

Therefore, in *Harpaz’s method*, the *RRR* was used instead of *confidence* as the second parameter to qualify the worthiness or strength of an association rule (Harpaz et al., 2010 ).

The *RRR* is defined as the ratio of the observation frequency of the rule to the prediction frequency of the baseline, and is shown as Eq. 43.

The other disproportionality analysis methods are based on the *RRR*, namely the *BCPNN* and the *EBGM* in the signal detection of a single drug.

(43) $$RRR=\frac{Observed}{Expected}=confidence\;\left(drug\;D_{1}\;and\;drug\;D_{2}\to AE\right)\;\times \;N$$

*N* is the total number of records in the data.

Extrapolating from Harpaz’s evaluation sample, the full set of potential DDIs identified by the method can be described by the taxonomy and proportions shown below.

Drugs are divided into the following three categories; (1) drugs known to be administered together or treat the same indication: 57%; (2) drugs with the same active ingredient: 2%; and (3) supposedly unrelated drugs: 41%.

AEs are divided into the following four categories: (1) one of the drugs is known to cause effect: 22%; (2) all drugs are known to cause effect: 21%; (3) none of the drugs is known to cause effect: 27%; and (4) confounded association, where drugs are administered to treat the AE: 30%.

The DDIs are divided into the following two categories: (1) known drug interaction: 35% and (2) unknown drug interaction: 65%.

In evaluations using *Harpaz’s method*, the results demonstrate that a significant number of DDIs can be identified. Additionally, the very low *p*-value indicates that it is extremely unlikely that Harpaz’s method detected them just by chance, and thus is a valid statistical model for signal detection.

#### Noguchi’s Method of Association Rule Mining Model

We proposed *Noguchi’s method* using the *association rule mining model* (Noguchi et al., 2018a). In *Noguchi’s method*, the antecedent of rule *X* was defined as *drug D*_2__(or__1)_ and the consequent of rule *Y* was defined as *drug D*_1__(or__2)_-induced AE. That is, *Noguchi’s method* focuses on how much additional *drug D*_2__(or__1)_ contributes to *drug D*_1__(or__2)_-induced AE.

(44) lift (drug D2(or1)→drug D1(or2)induced AE)=confidence (drug D2(or1)→drug D1(or2)induced AE)support (drug D1(or2)induced AE)

The *lift* according to this model indicates that the presence of *drug D*_2__(or__1)_ influences the probability of *drug D*_1__(or__2)_-induced AE. Furthermore, in this method, it was confirmed by *conviction* that the DDIs obtained are not a false prediction.

(45) conviction(drug D2(or1)→drug D1(or2)induced AE)=1−support(drug D1(or2)induced AE)1−confidence(drug D2(or1)→drug D1(or2)induced AE)

In the study by Noguchi et al., *lift* of >1 and *conviction* of >1 were used as the criterion for detection using the *association rule mining model*. As the risk data for verification was created by the *combination risk ratio model* presented in *Section 2.6*, there is no combination of n < 3 in the risk data for verification. Therefore, in the verification, the combination of n_111_ < 3 was excluded from the signal and n_111_ ≥ 3 was added to the criterion for detection.

*Noguchi’s method* has high detection power (sensitivity: 99.05%, specificity: 92.60%, Youden’s index: 0.917, positive predictive value: 78.57%, negative predictive value: 99.72% *F*-score: 0.876) like the *additive model* and *multiplicative model* (Noguchi et al., 2018a).

In *Noguchi’s method*, to compare the detection power, all combinations of DDIs were calculated using the *association rule mining model*. Therefore, it has not been determined how much computation time could be reduced compared to the previous methods using the *a priori algorithm*.

However, given the number of drugs registered in the spontaneous reporting systems, there are several potential combinations of DDIs. As *Noguchi’s method* simplifies the computation, it is expected that the time for signal detection will be reduced as well as statistical models using other *association rule mining model* in actual search.

The *association rule mining model* is easy to extend to higher-order interactions. However, among the three methods presented in this review, the gold-standard has not been determined.

The chi-squared statistics is useful to determine the statistical significance level. Alvarez showed that chi-square statistics can be calculated directly using *confidence*, *support*, and *lift* with Eq. 46 (Alvarez, 2003).

(46) $$\chi^{2}=T\times {\left(lift\right)}^{2}\times \frac{support\times confidence}{\left(confidence-support\right)\times \left(lift-confidence\right)}$$

The chi-squared statistics make it easy to validate combinations obtained using the standard *association rule mining model* (e.g., *Shirakuni’s method* and *Noguchi’s method*), and can identify statistically significant signals of DDIs that might be false positives.

### Causal Association Rule Discovery Model

As described in *Association Rule Mining Model*, the *association rule mining model* is often used to discover the signals of potential DDIs in the spontaneous reporting system. However, the main limitation of the traditional *association rule mining model* is that the strength of signals is measured based on correlation, not causality.

Several studies have been reported on the concept of causality, such as inductive causality models (Pearl, 2000), causal Bayesian network based methods (Spirtes et al., 2001), an additive noise model (Hoyer et al., 2008), and a hybrid approach (Cai et al., 2013), however causal discovery on high-order and sparse data of DDIs is still unsolved.

To solve this problem, instead of reconstructing a causal Bayesian network, Cai et al. proposed the *causal association rule discovery* (*CARD*) *model* with the aim to detect the true causal relationship between the concomitant use of two drugs and AEs (Cai et al., 2017).

For the rule *X* → *Y* with *X* ≥ 3, any sub-rules containing two antecedents must also form the V-structure with the AE: *drugs D*_1_ → AE ← *drugs D*_2_ (e.g., aspirin → Bleeding ← warfarin).

Because the interesting of rule *X* (*drugs D*_1_, *drugs D*_2_) → *Y* (AE) is dependent on the weakness of its sub-rules, and the *causal association interesting measure* (*CAIM*) is defined as follows:

(47) $$CAIM\;\left(X\to Y\right)=\underset{\left\{X_{i1},\;X_{i2}\right\}\;\subset \;Y}{\min}CAIM\;\left(X_{i1}X_{i2}\to Y\right)$$

The dominance of the *CARD model* was determined by physician assessment of 100 randomly selected higher-order associations detected using the *CARD model* and *Harpaz’s method of association rule mining model* (cf. *Harpaz’s Method of Association Rule Mining Model*) (Harpaz et al., 2010). In the identification of known DDI, the *CARD model* was more accurate than *Harpaz’s method*: *CARD model* (20%) vs. *Harpaz’s method* (10%). Furthermore, in the *CARD model*, the detection of unknown combinations is less than *Harpaz’s method*: *CARD model* (50%) vs. *Harpaz’s method* (79%) (Cai et al., 2017).

## Limitation

The spontaneous reporting systems used in these studies are based on clinical trials and post-marketing spontaneous reports, so only AEs observed are registered, and their causal relationship is unclear. Therefore, the cases may be underreported. Furthermore, the number of reports and signal values are influenced by various factors. Although not necessarily apparent, the number of cases increases in the first 2 years post-marketing and then begins to decrease. This is known as the Weber effect (Weber, 1984; Hartnell and Wilson, 2004).

The number and score of signals also possibly fluctuate during several years after launching (Hochberg et al., 2009). After drug-induced AE is highlighted, the number of reports may generally be accelerated. This is known as the notoriety effect (Pariente et al., 2007).

Additionally, the reports of drugs in the same class to those reported may also be accelerated. This is known as the ripple effect (Pariente et al., 2007).

The signal may be underestimated by numerous reports and that the same AE is associated with other drugs. This is called the masking effect or cloaking effect (Wang et al., 2010).

Matsuda et al. (2015) clarified that factors related to drug-induced AEs reporting attitudes in Japan may be different from those in other countries due to the involvement of medical representatives early post-marketing phase vigilance as a part of Japanese unique system of surveillance and the voluntary reporting process.

Thus, the spontaneous reporting systems are affected by several reporting biases and the state of the country’s survey. Furthermore, the report rates of AEs vary from year to year, and the value of the signal can easily vary with the timing of the survey.

In addition to the general limitations of study using the spontaneous reporting systems, the research of DDIs has some unique limitations.

In the surveillance for of DDIs, the lack of information about one of the two drugs will overestimate the *RRR* of drug-induced AEs, when either drug is used alone (Norén et al., 2008).

This is a serious problem in evaluating the AEs of DDIs, because it leads to under-reporting of *n*_111_ and over-reporting of *n*_101_ or *n*_011_ (**Figure 1**). Furthermore, some of these statistical models do not apply to interactions with three or more drugs.

Finally, these statistical models are designed to focus on the detection of synergism rather than antagonism among some interaction of DDIs.

## Conclusions and Perspectives

In this review, we have discussed statistical methodologies for signal detection of DDIs in spontaneous reporting systems. To the best of our knowledge, this is the latest review including recently proposed statistical methodologies.

The bivariate disproportionality analysis (e.g., single drug-induced AE) represents the bulk of daily routine of PhV. However, as the use of multiple drugs becomes more common, the problems of AEs due to DDIs cannot be ignored. Therefore, in the future operations of PhV, it is important to detect signals of unknown DDIs at an early stage.

In the bivariate disproportionality analysis, the frequentist methods generally have the following advantages and limitations compared with Bayesian methods. Several comparative studies of detection trends of these detection approaches have been reported (van, Puijenbroek et al., 2002; Kubota et al., 2004; Li et al., 2008; Bonneterre et al., 2012; Ang et al., 2016; Pham et al., 2019).

The advantages of the frequentist methods are generally as follows: 1. early signal detection, 2. sensitive, 3. easily applicable, and 4. easy to understand. While the limitations are 1. detection of false positive signals and 2. low specificity.

Although these advantages and limitations are considered to show a similar tendency in the signal detection models of DDIs, at this stage, the verification is not sufficient. Furthermore, the statistical models introduced in *Statistical Methodology* are not sufficiently clarified the difference in detection power. Therefore, in the future, it is necessary to examine the similarity and specificity of the signal detection tendency of each statistical model introduced.

As mentioned in *Limitation*, there are various biases (Weber, 1984; Hartnell and Wilson, 2004; Pariente et al., 2007; Hochberg et al., 2009; Wang et al., 2010; Matsuda et al., 2015) as these signals are calculated using the spontaneous reporting system. So the signal obtained is only a hypothesis. This does not change whether it is signals of single drug or DDIs. Therefore, considerable attention must also be paid to the interpretation of results in signal research of DDIs.

As indicated so far, most studies have focused on the analysis of AEs caused by the concomitant use of two drugs. However, in polypharmacy patients, the occurrence of AEs by interaction of multiple drugs (e.g., fourth order drug interaction: *drug D*_1_–*drug D*_2_–*drug D*_3_–*AE*) is a concern. Therefore, in the future, establishment of a signal detection method for this higher order drug interaction will be more important.

This review has introduced only statistical methodologies for detecting DDIs based on the number of AEs reported.

In recent years, the method for detecting the signals that use time-to-onset instead of the number of reports have been studied (van Holle et al., 2012; van Holle et al., 2014; Scholl and Van Puijenbroek, 2016), but there are no examples of using them for DDIs. Since it may be possible to detect the signals that cannot be obtained with the statistical models introduced in this review, further studies are expected.

## Author Contributions

YN and HT wrote the manuscript. TT also contributed with the paper organization. All the authors contributed with the bibliographic research.

## Funding

This study was supported by JSPS KAKENHI Grant Number 19K20731.

## Conflict of Interest

Although Laboratory of Community Healthcare Pharmacy, Gifu Pharmaceutical University, is financially supported by donations from WELCIA YAKKYOKU CO., LTD., the authors report no conflicts of interest regarding the content of this article. 

The handling editor and reviewer W-CC declared their involvement as co-editors in the Research Topic, and confirm the absence of any other collaboration.

## References

[B1] Agrawal R. Srikant R. (1994). “Fast algorithms for mining association rules,” in Proceedings of the 20th International Conference on Very Large Databases, vol. 487-499 (San Francisco, CA: Morgan Kaufman Publishers Inc.).

[B2] Agrawal R. Imielinski T. Swami A. N. (1993). Mining association rules between sets of items in large databases. ACM SIGMOD Int. Conf. Manage. Data, 207–216. 10.1145/170035.170072

[B3] Almenoff J. S. DuMouchel W. Kindman L. A. Yang X. Ram D. (2003). Disproportionality analysis using empirical Bayes data mining: a tool for the evaluation of drug interactions in the post-marketing setting. Pharmacoepidemiol Drug Saf. 12, 517–521. 10.1002/pds.885 14513665

[B4] Alvarez S. A. (2003). Chi-squared computation for association rules: preliminary results. Tech. Rep. BCCS-2003–01.

[B5] Ang P. S. Chen Z. Chan C. L. Tai B. C. (2016). Data mining spontaneous adverse drug event reports for safety signals in Singapore - a comparison of three different disproportionality measures. Expert Opin. Drug Saf. 15, 583–590. 10.1517/14740338.2016.1167184 26996192

[B6] Aronson J. K. (2004). Classifying drug interactions. Br. J. Clin. Phramacol 56, 343–344. 10.1111/j.1365-2125.2004.02244.x PMC1884601 15373925

[B7] Banda J. M. Callahan A. Winnenburg R. Strasberg H. R. Cami A. Reis B. Y. (2016). Feasibility of prioritizing drug-drug-event associations found in electronic health records. Drug Saf. 39, 45–57. 10.1007/s40264-015-0352-2 26446143 PMC4712252

[B8] Bate A. Lindquist M. Edwards I. R. Olsson S. Orre R. Lansner A. (1998). A Bayesian neural network method for adverse drug reaction signal generation. Eur. J. Clin. Pharmacol. 54, 315–321. 10.1007/s002280050466 9696956

[B9] Bonneterre V. Bicout D. J. Gaudemaris R. (2012). Application of pharmacovigilance methods in occupational health surveillance: comparison of seven disproportionality metrics. Saf. Health Work. 3, 92–100. 10.5491/SHAW.2012.3.2.92 22993712 PMC3440466

[B10] Cai R. Liu M. Hu Y. Melton B. L. Matheny M. E. Xu H. (2017). Identification of adverse drug-drug interactions through causal association rule discovery from spontaneous adverse event reports. Artif Intell Med. 76, 7–15. 10.1016/j.artmed.2017.01.004 28363289 PMC6438384

[B11] Cai R. Zhang Z. Hao Z. (2013). “SADA: a general framework to support robust causation discovery,” in Proceedings of the 30th International Conference on Machine Learning, vol. 28(2) 208-216.

[B12] Caster O. Noren G. N. Madigan D. Bate A. (2010). Large-scale regression-based pattern discovery: the example of screening the WHO global drug safety database. Stat. Analy. Data Min. 3, 197–208. 10.1002/sam.10078

[B13] DuMouchel W. Harpaz R. (2012). Regression-adjusted GPS algorithm (RGPS). Oracle White Paper November..

[B14] DuMouchel W. Pregibon D., (2001). “Empirical Bayes Screening for Multi-item Associations,” in Proceedings of the Seventh ACM SIGKDD International Conference on Knowledge Discovery and Data Mining, KDD‘01, 67-76 (New York, NY, USA: ACM).

[B15] Evans S. J. Waller P. C. Davis S. (2001). Use of proportional reporting ratios (PRRs) for signal generation from spontaneous adverse drug reaction reports. Pharmacoepidemiol. Drug Saf. 10, 483–486. 10.1002/pds.677 11828828

[B16] Genkin A. Lewis D. D. Madigan D. (2007). Large-scale Bayesian logistic regression for text categorization. Technometrics 49, 291-304 10.1198/004017007000000245

[B17] Gosho M. (2018). Risk of hypoglycemia after concomitant use of antidiabetic, antihypertensive, and antihyperlipidemic medications: a database study. J. Clin. Pharmacol. 58, 1324–1331. 10.1002/jcph.1147 29762878

[B18] Gosho M. (2019). Rhabdomyolysis risk from the use of two-drug combination of antidyslipidemic drugs with antihypertensive and antidiabetic medications: a signal detection analysis. Fundam Clin. Pharmacol. 33, 339–346. 10.1111/fcp.12435 30575126

[B19] Gosho M. Maruo K. Tada K. Hirakawa A. (2017). Utilization of chi-square statistics for screening adverse drug-drug interactions in spontaneous reporting systems. Eur. J. Clin. Pharmacol. 73, 779–786. 10.1007/s00228-017-2233-3 28280890

[B20] Harpaz R. Chase H. S. Friedman C. (2010). Mining multi-item drug adverse effect associations in spontaneous reporting systems. BMC Bioinf. 11, S7. 10.1186/1471-2105-11-S9-S7 PMC2967748 21044365

[B21] Harpaz R. DuMouchel W. LePendu P. Bauer-Mehren A. Ryan P. Shah N. H. (2013). Performance of pharmacovigilance signal - detection algorithms for the fda adverse event reporting system. Clin. Pharmacol. Ther. 93, 539–546. 10.1038/clpt.2013.24 23571771 PMC3857139

[B22] Harpaz R. Haerian K. Chase H. S. Friedman C. (2010). Statistical mining of potential drug interaction adverse effects in fda’s spontaneous reporting system. AMIA Annu. Symp Proc. 2010, 281–285. 21346985 PMC3041376

[B23] Hartnell N. R. Wilson J. P. (2004). Replication of the Weber effect using postmarketing adverse event reports voluntarily submitted to the United States Food and Drug Administration. Pharmacotherapy 24, 743–749. 10.1592/phco.24.8.743.36068 15222664

[B24] Hochberg A. M. Hauben M. Pearson R. K. O’Hara D. J. Reisinger S. J. (2009). Systematic investigation of time windows for adverse event data mining for recently approved drugs. J. Clin. Pharmacol. 49, 626–633. 10.1177/0091270009333484 19451402

[B25] Hoyer P. O. Janzing D. Mooij J. Peters M. Scholkopf B. (2008). “Nonlinear causal discovery with additive noise models,” in Neural Information Processing Systems (NIPS), vol. 689-696 (Vancouver, Canada).

[B26] Koski T. Orre R., (1998). “Statistics of the information component in Bayesian neural networks,” in Technical Report (Stockholm, Sweden: department of numerical analysis and computing science, royal institute of technology).

[B27] Kubota K. (2001). Signal detection from spontaneous reports - new Methods in MCA in the UK, FDA in the US and WHO. Jpn. J. Pharmacoepidemiol. 6, 101–108. 10.3820/jjpe1996.6.101

[B28] Kubota K. Koide D. Hirai T. (2004). Comparison of data mining methodologies using Japanese spontaneous reports. Pharmacoepidemiol Drug Saf. 13, 387–394. 10.1002/pds.964 15170768

[B29] Li C. Xia J. Deng J. Jiang J. (2008). A comparison of measures of disproportionality for signal detection on adverse drug reaction spontaneous reporting database of Guangdong province in China. Pharmacoepidemiol Drug Saf. 17, 593–600. 10.1002/pds.1601 18432629

[B30] Matsuda S. Aoki K. Kawamata T. Kimotsuki T. Kobayashi T. Kuriki H. (2015). Bias in spontaneous reporting of adverse drug reactions in Japan. PloS One 10, e0126413. 10.1371/journal.pone.0126413 25933226 PMC4416713

[B31] Mizuno T. Umemura T. Sakai T. Fukatsu M. Yamada T. Kajiguchi T. (2016). Signal detection on the concomitant use of deferasirox with other drugs and acute renal failure using data mining of the Japanese adverse drug event report database and evaluation by a case-control study. Jpn. J. Pharm. Health Care Sci. 42, 717–726. 10.5649/jjphcs.42.717

[B32] Noguchi Y. Esaki H. Asano S. Yokoi T. Usui K. Kato M. (2015). Analysis of effects of the diuretics on levels of blood potassium and blood sodium with angiotensin receptor blockers and thiazide diuretics combination therapy: data mining of the Japanese adverse drug event report database, JADER. Jpn. J. Pharm. Health Care Sci. 41, 488–496. 10.5649/jjphcs.41.488

[B33] Noguchi Y. Katsuno H. Ueno A. Otsubo M. Yoshida A. Kanematsu Y. (2018c). Signals of gastroesophageal reflux disease caused by incretin-based drugs: a disproportionality analysis using the Japanese adverse drug event report database. J. Pharm. Health Care Sci. 4, 15. 10.1186/s40780-018-0109-z 29946474 PMC6004661

[B34] Noguchi Y. Ueno A. Katsuno H. Otsubo M. Yoshida A. Kanematsu Y. (2018b). Analyses of non-benzodiazepine-induced adverse events and prognosis in elderly patients based on the Japanese adverse drug event report database. J. Pharm. Health Care Sci. 4, 10. 10.1186/s40780-018-0106-2 29760940 PMC5937044

[B35] Noguchi Y. Ueno A. Otsubo M. Katsuno H. Sugita I. Kanematsu Y. (2018a). A new search method using association rule mining for drug-drug interaction based on spontaneous report system. Front. Pharmacol. 9, 197. 10.3389/fphar.2018.00197 29593533 PMC5854950

[B36] Norén G. N. Bate A. Orre R. Edwards I. R. (2006). Extending the methods used to screen the WHO drug safety database towards analysis of complex associations and improved accuracy for rare events. Stat. Med. 25, 3740–3757. 10.1002/sim.2473 16381072

[B37] Norén G. N. Sundberg R. Bate A. Edwards I. R. (2008). A statistical methodology for drug-drug interaction surveillance. Stat. Med. 27, 3057–3070. 10.1002/sim.3247 18344185

[B38] Orre R. Lansner A. Bate A. (2000). Lindquist M. Bayesian neural networks with confidence estimations applied to data mining. Comput. Stat. Data Anal. 34, 473–493. 10.1016/S0167-9473(99)00114-0

[B39] Pariente A. Gregoire F. Fourrier-Reglat A. Haramburu F. Moore N. (2007). Impact of safety alerts on measures of disproportionality in spontaneous reporting databases: the notoriety bias. Drug Saf. 30, 891–898. 10.2165/00002018-200730100-00007 17867726

[B40] Pearl J. (2000). Causality: models, reasoning and inference First edition. ed: Cambridge Univ Press.

[B41] Pham M. Cheng F. Ramachandran K. (2019). A comparison study of algorithms to detect drug-adverse event associations: frequentist, Bayesian, and Machine-Learning Approaches. Drug Saf. 42, 743–750. 10.1007/s40264-018-00792-0 30762164

[B42] Pirmohamed M. Orme M. (1998). “Drug interactions of clinical importance,” in Davies’s Textbook of Adverse Drug Reactions, Eds. Davies D. Ferner R. de Glanville H. (London: Chapman & Hall Medical), 888–912.

[B43] Qian Y. Ye X. Du W. Ren J. Sun Y. Wang H. (2010). A computerized system for detecting signals due to drug-drug interactions in spontaneous reporting systems. Br. J. Clin. Pharmacol. 69, 67–73. 10.1111/j.1365-2125.2009.03557.x 20078614 PMC2830599

[B44] Scholl J. H. Van Puijenbroek E. P. (2016). The value of time-to-onset in statistical signal detection of adverse drug reactions: a comparison with disproportionality analysis in spontaneous reports from the Netherlands. Pharmacoepidemiol Drug Saf. 25, 1361–1367. 10.1002/pds.4115 27686554

[B45] Shirakuni Y. Okamoto K. Kawashita N. Yasunaga T. Takagi T. (2009). Signal detection of drug complications applying association rule learning for Stevens-Johnson Syndrome. J. Com. Aid. Chem. 10, 118–127. 10.2751/jcac.10.118

[B46] Spirtes P. Glymour C. Scheines R. Cusation, Prediction, and Search Second Edition ed., 2001.

[B47] Susuta Y. Takahashi Y. (2014). Safety risk evaluation methodology in detecting the medicine concomitant use risk which might cause critical drug rash. Jpn. J. Pharmacoepidemiol. 19, 39–49. 10.3820/jjpe.19.39

[B48] Szarfman A. Machado S. G. O’Neill R. T. (2002). Use of screening algorithms and computer systems to efficiently signal higher-than-expected combinations of drugs and events in the US FDA’s spontaneous reports database. Drug Saf. 25, 381–392. 10.2165/00002018-200225060-00001 12071774

[B49] Thakrar B. T. Grundschober S. B. Doessegger L. (2007). Detecting signals of drug-drug interactions in a spontaneous reports database. Br. J. Clin. Pharmacol. 64, 489–495. 10.1111/j.1365-2125.2007.02900.x 17506784 PMC2048563

[B50] van Holle L. Tavares Da Silva F. Bauchau V. (2014). Signal detection based on time-to-onset: extending a new method from spontaneous reports to observational studies. Pharmacoepidemiol Drug Saf. 23, 849–858. 10.1002/pds.3669 24946233

[B51] van Holle L. Zeinoun Z. Bauchau V. Verstraeten T. (2012). Using time-to-onset for detecting safety signals in spontaneous reports of adverse events following immunization: a proof of concept study. Pharmacoepidemiol Drug Saf. 21, 603–610. 10.1002/pds.3226 22383219

[B52] van, Puijenbroek E. P. Bate A. Leufkens H. G. Lindquist M. Orre R. Egberts A. C. (2002). A comparison of measures of disproportionality for signal detection in spontaneous reporting systems for adverse drug reactions. Pharmacoepidemiol Drug Saf. 11, 3–10. 10.1002/pds.668 11998548

[B53] van, Puijenbroek E. P. Egberts A. C. Heerdink E. R. Leufkens H. G. (2000). Detecting drug-drug interactions using a database for spontaneous adverse drug reactions: an example with diuretics and non-steroidal anti-inflammatory drugs. Eur. J. Clin. Pharmacol. 200056, 733–738. 10.1007/s002280000215 11214785

[B54] van, Puijenbroek E. P. Egberts A. C. Meyboom R. H. Leufkens H. G. (1999). Signalling possible drug-drug interactions in a spontaneous reporting system: delay of withdrawal bleeding during concomitant use of oral contraceptives and itraconazole. Br. J. Clin. Pharmacol. 47, 689–693. 10.1046/j.1365-2125.1999.00957.x 10383548 PMC2014250

[B55] Wang H. W. Hochberg A. M. Pearson R. K. Hauben M. (2010). An experimental investigation of masking in the US FDA adverse event reporting system database. Drug Saf. 33, 1117–1133. 10.2165/11584390-000000000-00000 21077702

[B56] Weber J. (1984). Epidemiology of adverse reactions to nonsteroidal anti-inflammatory drugs. Adv. Inflammation Res. 6, 1–7.

[B57] Yang X. Fram D. (2004). Using disproportionality analysis as a tool to explore drug-drug interavtions in AERS database. Pharmacoepidemiol. Drug Saf. 13, S247. 10.1002/pds.885 14513665

[B58] Yates F. (1934). Contingency tables involving small numbers and the χ^2^ test. Suppl. J. R Stat. Soc. 1, 217–235. 10.2307/2983604

